# A New Type I Peritrophic Membrane Protein from Larval *Holotrichia oblita* (Coleoptera: Melolonthidae) Binds to Chitin

**DOI:** 10.3390/ijms15046831

**Published:** 2014-04-22

**Authors:** Xiaomin Liu, Jie Li, Wei Guo, Ruijun Li, Dan Zhao, Xinna Li

**Affiliations:** 1 Institute of Cereal and Oil Crops, Hebei Academy of Agriculture and Forestry Sciences, Shijiazhuang 050035, Hebei, China; E-Mail: xiaominliu1981@gmail.com; 2 Plant Science and Technology College, Beijing University of Agriculture, Beijing 102206, China; 3 College of Plant Protection, Agricultural University of Hebei/Biological Control Centre of Plant Pathogens and Plant Pests of Hebei Province, Baoding 071001, Hebei, China; E-Mails: liruijun@163.com (R.L.); zhaodan@163.com (D.Z.); yjihn@163.com (X.L.); 4 Shijiazhuang Development and Reform Commission, Shijiazhuang 050011, Hebei, China; E-Mail: lijie@163.com

**Keywords:** peritrophic membrane, *Holotrichia oblita*, cDNA expression library, chitin binding protein, HoCBP76

## Abstract

Peritrophic membranes (PMs) are composed of chitin and protein. Chitin and protein play important roles in the structural formation and function of the PM. A new type I PM protein, HoCBP76, was identified from the *Holotrichia oblita*. HoCBP76 was shown as a 62.3 kDa protein by SDS-PAGE analysis and appeard to be associated with the PM throughout its entire length. In *H. oblita* larvae, the midgut is the only tissue where HoCBP76 could be detected during the feeding period of the larvae. The predicted amino acid sequence indicates that it contains seven tandem chitin binding domains belonging to the peritrophin-A family. HoCBP76 has chitin binding activity and is strongly associated with the PM. The HoCBP76 was not a mucin-like glycoprotein, and the consensus of conserved cysteines appeared to be CX_13–17_CX_5_CX_9_CX_12_CX_7_C. Western blot analysis showed that the abundance of HoCBP76 in the anterior, middle and posterior regions of the midgut was similar, indicating that HoCBP76 was secreted by the whole midgut epithelium, and confirmed the *H. oblita* PM belonged to the Type I PM. Immunolocalization analysis showed that HoCBP76 was mainly localized in the PM. The HoCBP76 is the first PM protein found in the *H. oblita*; however, its biochemical and physiological functions require further investigation.

## 1. Introduction

At one or more stages of the life cycle in most insects, peritrophic membranes (PMs) surround the food bolus in the digestive tract, facilitate food digestion and provide protection for midgut epithelial cells from invasion by viruses, bacteria and other pathogens. PMs are primarily composed of chitin and proteins. Peritrophins, the most abundant PM proteins, are strongly bound to the PM, and can be solubilized by strong denaturants such as 6 M urea or 6 M guanidine HCl [1–3]. Many peritrophin proteins analyzed to date contain the peritrophin-A domains, which contain one or more putative chitin-binding domains (CBDs) consisting of a register of six cysteine residues, whereas the peritrophin-B and peritrophin-C domains contain 8 and 10 cysteine residues, respectively. The peritrophin-A domains are mainly present in peritrophins from the Diptera and Lepidoptera [2,4], but have also been found in some insect mucins [5].

Based on the PMs synthesis site, insect PMs are generally characterized into two types. Type I PM is secreted by the whole midgut epithelium [2,6]. This type of PM is studied mainly in the laval Lepidoptera and adult hematophagous Diptera, it has also been found in Coleoptera, Dictyoptera, Ephemeroptera, Hymenoptera, Odonata, Orthoptera and Phasmida [3,7–9]. There have been descriptions of the structure and formation of Coleoptera larval PM at the microscopic level [10]. For the Coleoptera larval *Gibbium psylloides*, its posterior midgut can extrude a PM-like material which spins a cocoon [11]. Type II PM is formed by the cardia, which is the specialized tissue at the entrance of the midgut. It has been found in Dermaptera, Isoptera, Embiodea, some Lepidoptera, and the larvae of Diptera [3,12]. Despite the different modes by which type I and type II PM are synthesized, their protein components are similar. Estimates of the number of PM proteins vary widely, from a few to many dozen. Tellam proposed four classes of PM proteins based on the ease with which they can be removed from the PM [2]. Much research has been directed toward a group of integral Class III PM proteins collectively known as peritrophins.

Both of the two types of PM are composed of chitin, prtoteins and proteoglycans. Chitin contributes only a small proportion of PM, but is considered to be a significant structural component. Chemical analysis showed that chitin contents of insect PMs ranged from 3.7% to 12.9% [13,14]. Chitin microfibrils are believed to form a strong and elastic mesh-like network for PM assembly. Analysis of numerous insect species reveals that PM proteins always have multiple chitin-binding domains, which are proposed to be involved in maintaining the network structure of PM chitin fibrils. The invertebrate CBDs are believed to evolve from a common ancestor, and are predicted not to have any secondary structure. The conserved cysteine-mediated disulfide bonds are suggested to stabilize the folding, and the conserved aromatic residues are thought to be involved in saccharide binding [15]. The invertebrate CBD was first characterized in the peritrophic membrane protein peritrophin-44 from *Lucilia cuprina*, which was confirmed to bind to chitin within the PM [16]. It has been described how the removal of the CBD from some chitin-binding proteins could decrease the affinity for chitin, while addition of one or more domains could increase the affinity for chitin [17,18].

To date, about 20 PM proteins have been identified and characterized from different insects [7]. In spite of the reviewed studies on different insect PMs, there is no data on the *Holotrichia oblita* (Coleoptera: Melolonthidae) PM. In this paper, we report the identification of a new type I peritrophic membrane protein, HoCBP76, from the *H. oblita*. HoCBP76 possesses severn six-cysteine-containing chitin-binding domains related to the peritrophin A domain and exhibits a strong chitin-binding activity, so it belongs to the PM protin Class III.

## 2. Results and Discussion

### 2.1. Identification of the cDNA Coding for a New H. oblita PM Protein, HoCBP76

**Figure 1.**
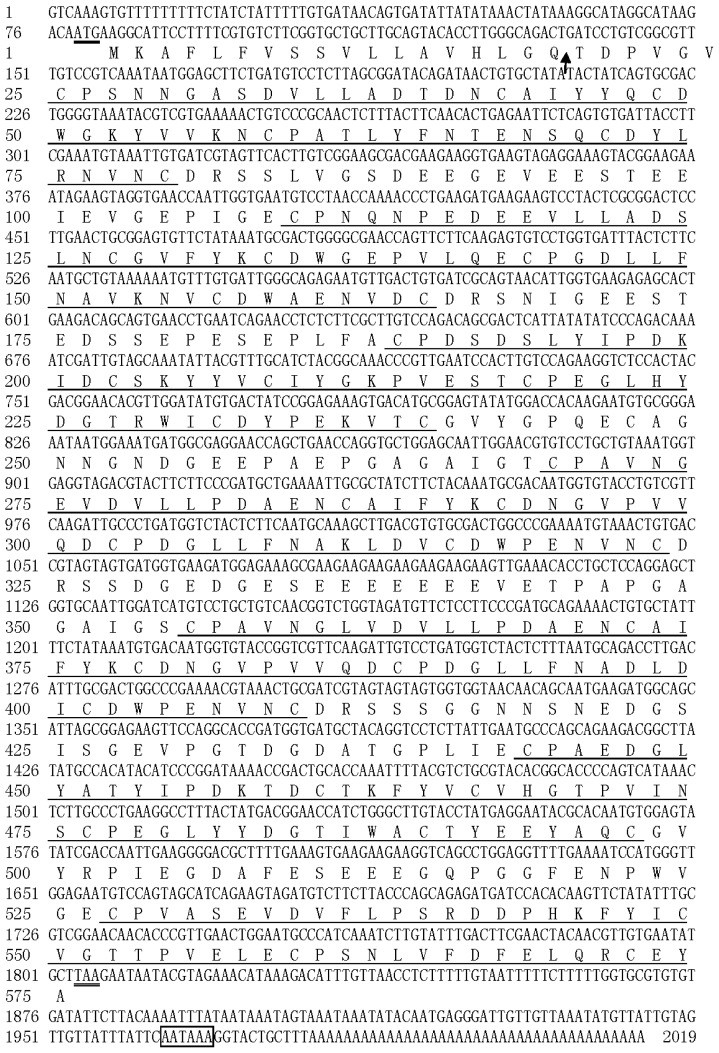
Nucleotide sequence of the cDNA for HoCBP76 and its deduced amino acid sequence (GenBank™ accession number: HM596340). The translation initiation codon ATG and stop codon TAA are double underlined. The predicted signal peptide cleavage site is indicated by a vertical arrow. The potential polyadenylation signal sequence is marked in a box. Eight chitin binding domains are underlined from *N*- to *C*-terminus of the protein.

By immunoscreening of the *H. oblita* midgut cDNA expression library with antibodies against a collection of *Helicoverpa armigera* midgut PM proteins, 254 positive cDNA clones were obtained and sequenced. A full-length cDNA clone was identified and designated as *HoCBP76* (GenBank Accession No. HM596340). The cDNA is 2019 bp in length, containing an ORF of 1725 bp, including a 5′ untranslated region of 78 bp and a 3′ untranslated region of 216 bp, and a putative polyadenylation signal (AATAAA) located at 11 bp upstream of the polyA tail (Figure 1). The deduced protein sequence showed that HoCBP76 was synthesized as a preprotein of 575 amino acid residues with the predicted molecular weight of 62.3 kDa, pI of 3.51 and a 19-amino acid signal peptide predicted by the software SignaIP [19]. The secreted HoCBP76 contains one potential *O*-glycosylation site at Thr^344^ indicating that the HoCBP76 was not a mucin-type PM protein and one putative *N*-glycosylation site based on the presence of the sequence pattern Asn–Xaa–Thr/Ser located at amino acid residues from 417 to 419 [20,21]. HoCBP76 contains seven tandem putative chitin binding domains which belonged to the peritrophin-A domains.

**Figure 2.**
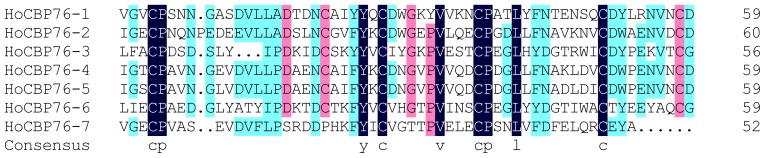
Alignment of the predicted chitin binding domain sequences from HoCBP76 protein. The conserved amino acids are shaded. The consensus sequence is shown at the bottom.

PMs are present in most insects, except for Hemiptera and Thysanoptera, which have perimicrovillar membranes in their midgut cells [6]. The most studied PM proteins were identified from Lepidoptera insects, and fewer PM proteins were characterized from Colepotera insects. In this study, we identified a novel PM chitin binding protein HoCBP76 from *H. oblita* (Coleoptera: Melolonthidae) larvae. Alignment of the peritrophin-A domains from the HoCBP76 was shown in Figure 2, the result showed that the six cysteine residues within the domains were well conserved from 1 to 6 CBD. The consensus of conserved cysteines appeared to be CX_13–17_CX_5_CX_9_CX_12_CX_7_C (where X is any amino acid except cysteine), which are similar to the predicted chitin binding sequences from PM proteins of *Tribolium castaneum* and other species [22]. Conserved aromatic amino acids were located between Cys2 and Cys3, Cys5 and Cys6, which was thought to be involved in the binding of sugars because other sugar-binding motifs called hevein domains also contained conserved aromatic residues [23,24]. The seventh CBD of HoCBP76 had only four cysteines, which is similar to the peritrophic membrane protein PMP14 (GeneBank accession number GU128106) from *Tribolium castaneum* (Coleoptera: Tenebrionidae).

### 2.2. Identification of HoCBP76 from the H. oblita PM

**Figure 3.**
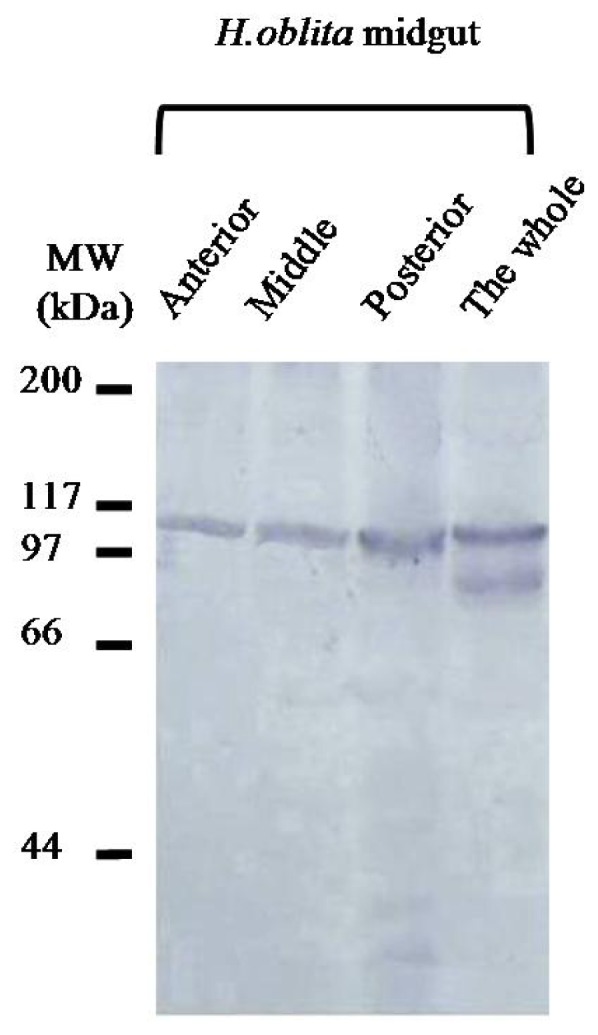
Identification of HoCBP76from *H. oblita* larval midgut proteins by western blot analysis with antibodies specific to HoCBP76. Proteins were from the anterior, middle, posterior regions of the *H. oblita* midgut epithelium and the whole midgut epithelium.

Antibodies reacting to HoCBP76 recognized an identical molecular weight protein from the *H. oblita* midgut proteins by Western blot analysis (Figure 3). The result showed that the abundance of HoCBP76 in the anterior, middle and posterior regions of the midgut was similar, indicating that HoCBP76 was secreted by the whole midgut epithelium, but was not by the cells at the entrance of the midgut (cardia). Western blot analysis confirmed that secreted the *H. oblita* PM belonged to the Type I PM, which was similar to the observations made in other Coleoptera insects, the *Diabrotica undecimpunctata* (Coleoptera: Chrysomelidae), which PM was synthesized along the length of the midgut epithelium, and secreted into the interstices between the microvilli of the brush border [11].

### 2.3. Localization of HoCBP76 in H. oblita Larvae

Western blot analysis of proteins from various tissues isolated from third instar *H. oblita* larvae showed that in addition to the PM, HoCBP76 was detected in midgut tissue but was not detectable from the larvalintegument, digestive fluid, Malpighian tubules, fat body and exuvia. In proteins from the PM, a main band and a number of minor bands with lower molecular weights were detected by Western blot ananlysis. The minor bands could be degradation products of HoCBP76. The expression showed some similar with that of TnPM-P42 protein, another PM protein without peritrophin domains in *Trichoplusia ni*. The TnPM-P42 protein was only expressed in the PM of midgut, not in hemolymph, Malpighian tubules, salivary glands, fat body, or integument [25].

### 2.4. Binding of Recombinant HoCBP76 to Chitin

**Figure 4.**
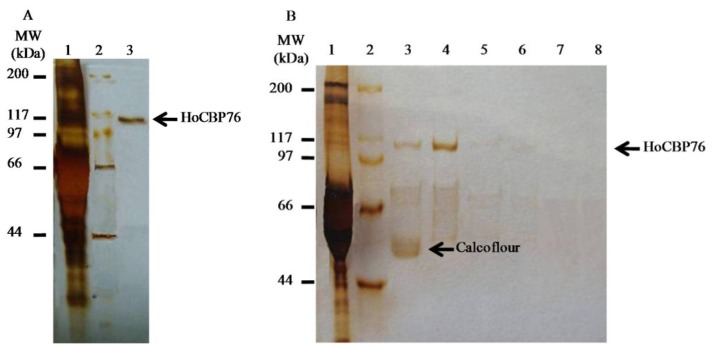
SDS-PAGE analysis of chitin binding activity of recombinant HoCBP76. (**A**) Silver staining analysis of recombinant HoCBP76 expressed in insect cells using recombinant baculovirus. Lane 1: culture media from HighFive cells infected with vHoCBP76, showing that the recombinant HoCBP76 was secreted into the medium. Lane 2: protein marker. Lane 3: chitin bound protein from the media of vHoCBP76 infected HighFive cells; (**B**) Silver staining analysis of recombinant HoCBP76 dissociated from the HoCBP76/regenerated chitin complex by different treatment. Lane 1: culture media from HighFive cells infected with vHoCBP76. Lane 2: protein marker. Lane 3–8: HoCBP76/regenerated chitin complex by incubation with 1% Calcoflour, 6 M Urea, 2% SDS + 5% β-ME, 2% SDS, PBS and 1 M NaCl, respectively.

HoCBP76 was expressed successfully in insect cells (Tn-5B1-4) with the recombinant baculoviruses. The recombinant protein, which was secreted into the cell culture medium, exhibited its activity to bind chitin (Figure 4A). The apparent molecular weight for recombinant HoCBP76 was more than 100 kDa, significantly higher than its predicted molecular weights of 62.3 kDa. This phenomenon was similar to PM proteins identified from other species [2,5,26]. The reason is probably because that *O*-Glycosylation of serine and threonine residues which is believed to increase the length of the polypeptide, making the structure of the glycosylated region rod-like by restricting the flexibility of the peptide backbone. This phenomenon was similar to PM proteins identified from other species, such as *C. bezziana*, *Trichoplusia ni*, and so on [2,5,26]. Subsequent chitin binding assay demonstrated that the recombinant HoCBP76 had chitin binding affinity (Figure 4B). The HoCBP76 tightly bounded to chitin and did not dissociate from the chitin following treatment with PBS, and 1 M NaCl. The protein was only partially dissociated with 2% SDS in the presence of 5% β-mercaptoethanol and 2% SDS. However, it was solubilized from the bound chitin by 6 M urea or by 1% Calcofluor.

**Figure 5.**
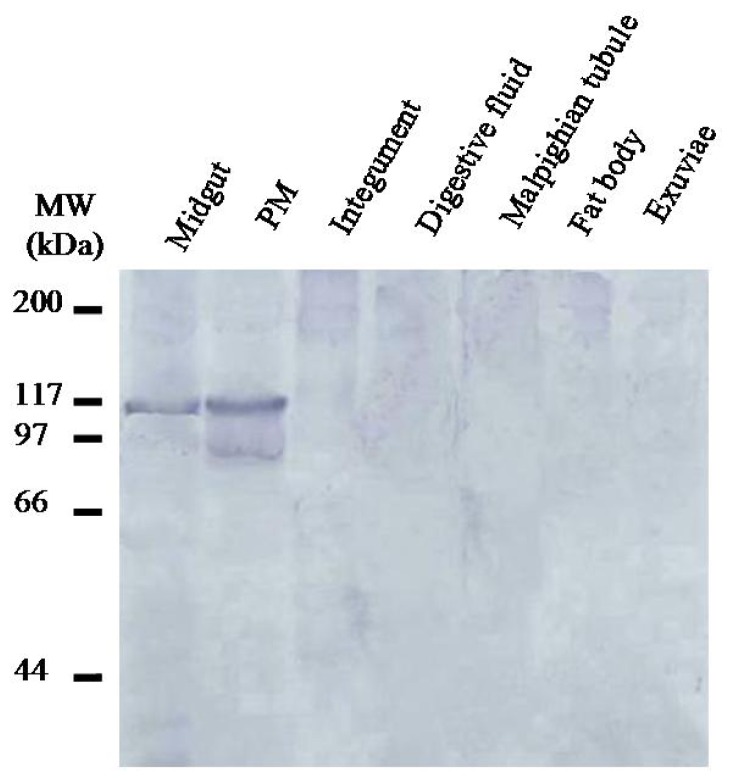
Detection of HoCBP76 in various *H. oblita* tissue and structure samples by western blot analysis.

The PM acts as a barrier against bacterial pathogens, but ingestion of insecticidal lectins, the insecticidal toxin from *Xenorhabdus nematophila*, plant cysteine proteases, the baculovirus metalloprotease enhancing and Cry toxins can destroy the PM [27,28]. To date, PM proteins identified from different insect species are all chitin binding proteins with or without mucin domains and may contain up to six putative chitin binding domains [29–31]. In this study, the vitro chitin-binding assays experimentally confirmed the strong chitin-binding affinity of HoCBP76 (Figure 4). The HoCBP76 had seven tandem putative chitin binding domains belonging to the peritrophin-A family, and was mainly localized in PM (Figure 5), which has been suggested to be a mechanism for PM formation [32].

## 3. Experimental Section

### 3.1. Insect Larvae and Collection of Larval Tissues

All the adults *Holotrichia oblita* were collected from Baoding, China. Larvae of *H. oblita* was reared in the laboratory condition at room temperature, soil humidity 18%. The third instar larvae were used for dissection to isolate the PMs and various tissues for analyses.

### 3.2. Construction and Immunoscreening of a cDNA Expression Library

Midgut total RNA was isolated from fresh midgut epithelial tissues of 3rd instar *H. oblita* larvae using the RNeasy mini kit from QIAGEN (Valencia, CA, USA), and poly(A)^+^ RNA was prepared with the Oligotex mRNA kit (Qiagen, Valencia, CA, USA). The cDNA expression library was constructed using ZAP cDNA synthesis and GigapackIII gold cloning kit (Stratagene, La Jolla, CA, USA). Starting with about 5 μg of poly(A)^+^ RNA, and the first-strand cDNA synthesis was performed using an oligo(dT) primer with an internal *Xho*I site and 5-methyl dCTP, after the second-strand cDNA synthesis, the cDNA was ligated to *Eco*RI adaptor and digested with *Xho*I, and then the size-fractionated cDNA was cloned directionally into the bacteriophage expression Uni-ZAP XR vector, packaged into phage particles and used to transfect *Escherichia coli*, resulting in 5 × 10^6^ recombinant plaques per μg of vector in the library. The screening procedure was according to the picoBlue™ immunoscreening kit manufacture’s specifications. The antibody used for screening was antiserum that was raised in rabbits against a purified PM proteins from *Helicoverpa armigera* (Lepidoptera: Noctuidae). The secondary antibody (goat anti-rabbit immunoglobulin, Sigma, St. Louis, MO, USA) was used at a dilution of 1:30,000. Finally the reactive cDNA clones were processed to rescue the pBluescript SK(−) phagemids by *in vivo* excision following the manufacture above described.

### 3.3. Recombinant HoCBP76 Expression

To express HoCBP76 in insect cells, a recombinant baculovirus was constructed, using the Bac-to-Bac baculovirus expression system (Invitrogen, Carlsbad, CA, USA). The cDNA for HoCBP76 was excised from the cDNA clone in pBluescript by digestion with *Eco*RI and *Xho*I, and cloned into the transfer plasmid vector pFASTBac1. The recombinant baculovirus with the cDNA for HoCBP76 was generated by transfection of bacmid DNA into BTI-Tn-5B1-4 insect cells and maintained in TNM-FH medium supplemented with 10% fetal bovine serum. The cell culture medium containing the secreted recombinant HoCBP76 was collected at 72 h post-infection.

### 3.4. Regenerated Chitin Preparation and Chitin-Binding Assay

Regenerated chitin for the chitin-binding assay was prepared from chitosan (Sigma Corporation, St. Louis, MO, USA) by the method of Molano *et al.* [33]. One gram of chitosan is ground in a mortar while adding slowly and in small portions 20 mL of 10% acetic acid, and is allowed to stand overnight at room temperature. The next day, 90 mL of methanol are added slowly with mixing and the cloudy solution is filtered. The filtrate is placed in a beaker on a magnetic stirrer and 1.5 mL of acetic anhydride were added. After about 1 min, the mixture gels. The gel is allowed to stand for about 30 min and then is cut up into small pieces with a spatula. After covering with methanol the suspension is homogenized for 1 min at maximum speed. The finely dispersed chitin is filtered with a medium porosity sintered-glass funnel and is washed with water to neutrality. The chitin is resuspended in 0.02% sodium azide to a concentration of about 15 mg/mL.

The binding assay mixture contained 40 mg regenerated chitin and 1 mL HoCBP76 containing cell culture medium, and allowed HoCBP76 protein to bind to chitin at 4 °C in suspension for overnight in the presence of 1 mM EDTA and 1 mM phenylmethylsulfonyl fluoride. The regenerated chitin bound with HoCBP76 was washed three times with PBS, followed by centrifugations. Aliquots of the resulting chitin bound with HoCBP76 were incubated with 1% Calcoflour, 6 M urea, 2% SDS + 5% β-mercaptoethanol, PBS, 2% SDS and 1 M NaCl, respectively. After 1 h incubation, the supernatants containing the HoCBP76 protein released from the chitin were collected and analyzed by silver-stained SDS-PAGE analysis.

### 3.5. Preparation of Antibodies Reacting to HoCBP76

A cDNA clone for HoCBP76 in pBluescript pHoCBP76 was used to produce the chitin-binding protein in the *E. coli* strain XL1-Blue (Strategene, La Jolla, CA, USA). To immobilize the solubilized proteins in the *E. coli* lysate, a piece of supported nitrocellulose membrane, Optitran BA-S85 (Schleicher and Schuell, Keene, NH, USA), was placed into the lysate in a Petri dish and incubated at room temperature for 1 h, followed by extensive washing five times with PBS and incubation in 3% BSA for 3 h. The nitrocellulose membrane then was incubated with a 100-fold dilution of the antiserum in PBS with 3% BSA at room temperature for 3 h to allow anti-HoCBP76 antibodies in the antiserum to bind to the blotted membrane. The membrane subsequently was washed five times with PBS, and finally the bound antibodies were eluted from the membrane by incubation in 5 mL of 0.1 M glycine buffer (pH 2.5) at room temperature for 10 min. The eluted antibody solution was collected, neutralized with 0.5 mL 1 M Tris-HCl buffer (pH 8.0).

### 3.6. Immunolocalization of HoCBP76 in H. oblita Larvae

All protein samples were analyzed by SDS-PAGE and electrophoretically transferred to nitrocellulose as described [25]. Tissues were dissected from mid-third instar larvae, exuviae from the larvae molting from second instar to third instar and fecal pellets from third instar larvae were also collected. Midgut digestive fluid was collected from mid-third instar larvae by stimulating the larval mouth parts in a test tube to induce regurgitation of the midgut fluid. Equal amounts of proteins from each tissue sample were mixed with 6× SDS-PAGE sample buffer (0.375 M Tris pH 6.8, 12% SDS, 60% glycerol, 0.6 M DTT, 0.06% bromophenol blue), boiled for 5 min, protein concentrations in the supernatants were estimated using the Bradford protein assay. One microgram of protein from each tissue extract, except for the PM extract, for which 0.04 mg of protein was used, was loaded onto the 10% SDS-polyacrylamide gel. Following electrophoresis, proteins were blotted onto Immobilon-P membranes (Millipore, Bedford, MA, USA), and then probed with antibodies reacting to HoCBP76 after treatment of the membrane with 3% BSA in PBS to block non-specific bindings, and bound antibodies were detected colorimetrically with alkaline phosphatase-conjugated goat anti-rabbit IgG and the bromochloroindolyl phosphate-nitro blue tetrazolium system.

## 4. Conclusions

A novel midgut peritrophic membrane (PM) protein HoCBP76 was identified from the *Holotrichia oblita* (Coleoptera: Melolonthidae). The presented results showed that HoCBP76 appeared to be associated with the PM throughout its entire length. HoCBP76 had chitin-binding activity and was strongly associated with the PM, which was similar to the currently known peritrophin type PM proteins. The identification of HoCBP76 in this study has provided the foundation for further investigation on the biochemical and physiological function of this new protein in the PM formation mechanism in Coleoptera insects.
